# MceG stabilizes the Mce1 and Mce4 transporters in *Mycobacterium tuberculosis*

**DOI:** 10.1016/j.jbc.2023.102910

**Published:** 2023-01-13

**Authors:** Rachael A. Fieweger, Kaley M. Wilburn, Christine R. Montague, Emma K. Roszkowski, Carolyn M. Kelly, Teresa L. Southard, Holger Sondermann, Evgeniya V. Nazarova, Brian C. VanderVen

**Affiliations:** 1 Microbiology & Immunology, College of Veterinary Medicine, Cornell University, Ithaca New York, USA; 2 Molecular Medicine, College of Veterinary Medicine, Cornell University, Ithaca New York, USA; 3 Biomedical Sciences; College of Veterinary Medicine, Cornell University, Ithaca New York, USA

**Keywords:** fatty acid transport, cholesterol, Mce transporter, Mce1, Mce4, MceG, LucA, ΔMceG, *mceG* in Mtb, ACN, acetonitrile, CFU, colony-forming unit, Mtb, *Mycobacterium tuberculosis*, RTS, real-time search, TEAB, triethylammonium bicarbonate, TMT, tandem mass tag, WCL, whole cell lysate

## Abstract

Lipids are important nutrients for *Mycobacterium tuberculosis* (Mtb) to support bacterial survival in mammalian tissues and host cells. Fatty acids and cholesterol are imported across the Mtb cell wall *via* the dedicated Mce1 and Mce4 transporters, respectively. It is thought that the Mce1 and Mce4 transporters are comprised of subunits that confer substrate specificity and proteins that couple lipid transport to ATP hydrolysis, similar to other bacterial ABC transporters. However, unlike canonical bacterial ABC transporters, Mce1 and Mce4 appear to share a single ATPase, MceG. Previously, it was established that Mce1 and Mce4 are destabilized when key transporter subunits are rendered nonfunctional; therefore, we investigated here the role of MceG in Mce1 and Mce4 protein stability. We determined that key residues in the Walker B domain of MceG are required for the Mce1- and Mce4-mediated transport of fatty acids and cholesterol. Previously, it has been established that Mce1 and Mce4 are destabilized and/or degraded when key transporter subunits are rendered nonfunctional, thus we investigated a role for MceG in stabilizing Mce1 and Mce4. Using an unbiased quantitative proteomic approach, we demonstrate that Mce1 and Mce4 proteins are specifically degraded in mutants lacking MceG. Furthermore, bacteria expressing Walker B mutant variants of MceG failed to stabilize Mce1 and Mce4, and we show that deleting MceG impacts the fitness of Mtb in the lungs of mice. Thus, we conclude that MceG represents an enzymatic weakness that can be potentially leveraged to disable and destabilize both the Mce1 and Mce4 transporters in Mtb.

*Mycobacterium tuberculosis* (Mtb) remains a major global health problem and current estimates indicate that tuberculosis claimed ∼1.4 million lives among HIV-negative individuals and caused ∼10 million new infections in the year 2021 alone (1). A defining feature of Mtb infections is that this bacterium persists in tissues for long durations while promoting the tissue pathology needed for dissemination and transmission between individuals (2). Thus, understanding how Mtb persists in host cells and or tissues could reveal new weaknesses that can be leveraged in drug development to better combat this pathogen.

The complete nutritional requirements of Mtb *in vivo* remain unknown (3), however, it is established that Mtb imports and metabolizes host-derived lipid nutrients to persist in host cells and tissues (4). Mtb has evolved specialized transporters that import nutrients across its lipid-rich cell wall that prevents diffusion of many macromolecules (5). Polar nutrients are imported through porin-like channels (5, 6, 7), while hydrophobic nutrients such as fatty acids and cholesterol are transported *via* the Mce1 and Mce4 transporters, respectively (8, 9). It is thought that Mce1 and Mce4 import substrates across the cell wall and deliver lipids directly to permease complexes embedded in the cytoplasmic membrane (4). Current models predict that the substrate-specific components of the Mce1 and Mce4 transporters (substrate-binding proteins and permeases) are encoded in two dedicated operons in the Mtb genome (10). Unlike most other transporter systems, Mce1 and Mce4 appear to use the shared ATPase, MceG, to power transport activity (8, 9, 11) and it is unclear how MceG coordinates ATP hydrolysis in association with these different transporters. Mce1 and Mce4 also share additional proteins to facilitate substrate import and/or stabilize the transporter complexes. For example, LucA facilitates cholesterol and fatty acid transport *via* the Mce1 and Mce4 transporters and also stabilizes the Mce1 transporter in Mtb (8, 12). Similarly, the Omam family of proteins facilitates cholesterol transport and stabilizes the Mce1 and Mce4 transporter homologs in *Mycobacterium smegmatis* (13, 14).

Given the growing evidence that proteins shared by Mce1 and Mce4 play a stabilizing role for the transporters, we sought to better understand how MceG facilitates Mce1- and Mce4-mediated transport and to determine if the ATPase activity of MceG could be corrupted in such a way to deactivate both Mce1 and Mce4. By deleting MceG or inactivating key residues in the Walker B motif of the protein, we found that components of the Mce1 and Mce4 transporters were destabilized and degraded. Lastly, we confirm that MceG is required for optimal bacterial fitness in the lungs of mice. Together, these studies establish that the MceG is a novel enzymatic weakness that is necessary for powering and stabilizing two different transporters. It is plausible that chemically inactivating MceG could simultaneously corrupt two important nutrient acquisition pathways that contribute to Mtb pathogenesis.

## Results

### The ATPase activity of MceG is required for fatty acid and cholesterol utilization

**Figure 1:**
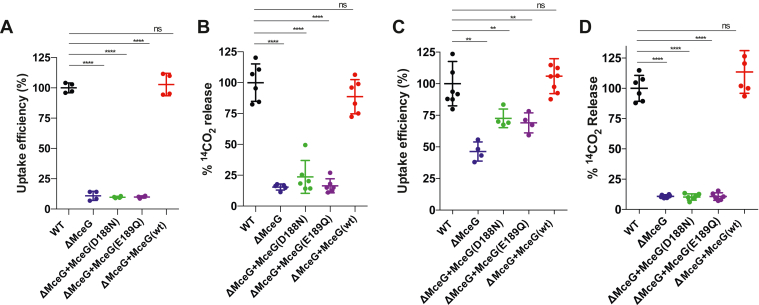
**The ATPase activity of MceG is required for the utilization of palmitic acid and cholesterol.***A*, whole cell quantification of the rate of palmitic acid import by Mtb cells. *B*, metabolic oxidation of palmitic acid to CO_2_. *C*, whole cell quantification of the rate of cholesterol import by Mtb cells. *D* catabolic release of CO_2_ from cholesterol. Data (n ≥ 4) ± SD. Significance was calculated using one-way ANOVA with Dunnett’s multiple comparisons test (∗∗*p* < 0.01) ns = not significant. To calculate the Uptake efficiency (%), bacteria were grown in media containing radiolabeled lipids and radioactive counts were measured from the cells over 2 h; these radioactive counts were used to calculate the rate of lipid uptake which was normalized to WT for each strain and expressed as a percentage (∗∗∗∗*p* < 0.0001). Mtb, *Mycobacterium tuberculosis.*

MceG conserves the canonical motifs needed for ATP binding, hydrolysis, and an ABC transporter family signature motif (15). Previously, it has been established that MceG is necessary for Mce4-mediated cholesterol transport in Mtb (9), and a conserved lysine residue in the Walker A motif of MceG (K66) is required for full virulence of Mtb in mice (11). Since it was not known if the putative active site residues of MceG are needed for substrate transport, we evaluated if the putative Walker B residues in MceG are indeed required for fatty acid and cholesterol import and metabolism. For this, we deleted *mceG* in Mtb (ΔMceG) by allelic exchange and complemented this strain by expressing WT MceG or variants of the protein where key Walker B residues have been mutated (D188N or E189Q). The bacteria express these variants of MceG from the native MceG promoter in a chromosomally integrating vector. Relative to WT or the complemented control, the ΔMceG mutant displayed a ∼90% reduction in the rate of ^14^C-palmitic acid import (Fig. 1*A*). Bacteria expressing the MceG Walker B variants displayed a similar reduction in the rate of fatty acid import (Fig. 1*A*). We also measured the metabolic oxidation of ^14^C-palmitic acid and found that there was an ∼85% reduction in the amount of fatty acid oxidized by the ΔMceG mutant relative to WT and the complemented control. Similarly, expressing the MceG Walker B variants failed to complement the ΔMceG mutant and displayed a similar reduction in the levels of fatty acid oxidation (Fig. 1*B*).

MceG is also required for cholesterol import and metabolism. Relative to WT or the complement control, the ΔMceG mutant had a ∼50% reduction in the rate of ^14^C-cholesterol import. The MceG Walker B variants did not fully complement the mutation and displayed ∼25% reduction in the rate of ^14^C-cholesterol import (Fig. 1*C*). Metabolism of ^14^C-cholesterol was also decreased in bacteria lacking MceG or in bacteria expressing the MceG Walker B variants. The ΔMceG mutant had a defect (∼90% decrease) in the ability to metabolize ^14^C-cholesterol relative to WT and the complemented control. The MceG Walker B variants failed to complement the ΔMceG mutant and displayed a similar reduction in the levels of cholesterol metabolism relative to the ΔMceG mutant (Fig. 1*D*).

The hydrophobic nature of the Mtb cell wall and cholesterol likely contribute to the relatively high background levels of ^14^C-cholesterol binding in the ΔMceG mutant using the cholesterol uptake assay (8, 9, 12). Thus, the defect in ^14^CO_2_ release likely better reflects the bacterium’s inability to import and metabolize the ^14^C-cholesterol substrate (Fig. 1*D*). Still, we sought to establish that nonspecific binding of cholesterol to the bacterial cells accounts for the high background levels of ^14^C-cholesterol binding in the uptake assay. It was previously reported that sodium azide effectively inhibits Mce4-mediated cholesterol transport in *Rhodococcus jostii* (16). In Mtb, we found that sodium azide effectively inhibited the metabolic conversion of ^14^C-cholesterol to ^14^C-CO_2_ (Fig. S1*A*), but this treatment did not decrease the relative rate of ^14^C-cholesterol binding in the ΔMceG cells (Fig. S1, *B* and *C*). Thus, even in the absence of MceG, there are substantial levels of ^14^C-cholesterol binding to the bacterial cells in this assay. It is unclear if the ^14^C-cholesterol nonspecifically interacts with the bacterial cell wall lipids or is captured by specific proteins. While this assay has relatively high background levels, it is still sensitive enough to detect an uncoupling of transport from bacterial metabolism of 14C-cholesterol. For example, Mtb mutants lacking Mam4B transport cholesterol to WT levels, but these bacteria are unable to metabolize ^14^C-cholesterol (8).

### The ΔMceG mutant is resistant to fatty acid intoxication

**Figure 2:**
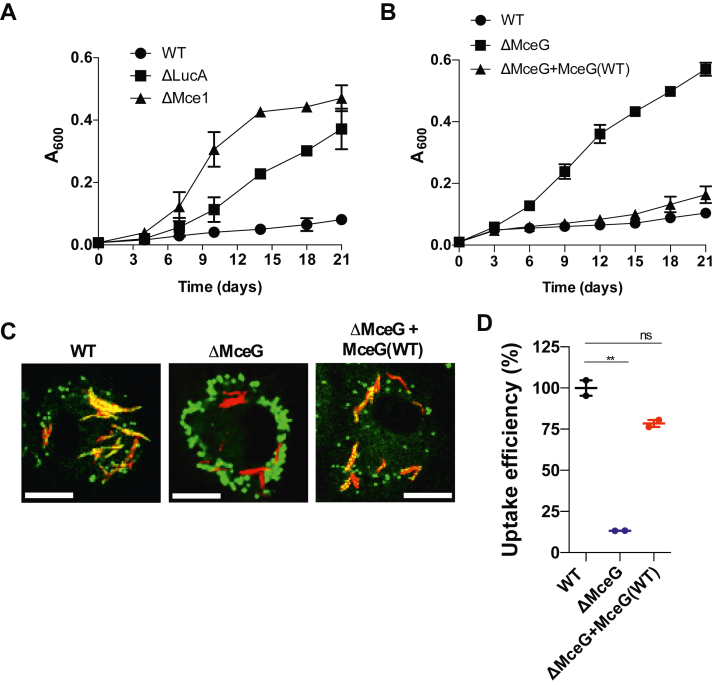
**MceG is required for fatty acid intoxication and for importing fatty acids during infection in macrophages.***A*, inhibition of Mtb growth in minimal media containing 25 μM palmitate (C16) occurs when the bacteria express a functional Mce1 transporter. *B*, the growth of Mtb lacking MceG is not inhibited in minimal media containing 25 μM palmitate (C16) without albumin. *C*, confocal microscopy analysis reveals that Bodipy-C16 does not accumulate in ΔMceG mutant as cytosolic lipid inclusions. Representative confocal images of infected macrophages (*red* = mCherry Mtb, *green* = Bodipy-C16). The scale bar represents 10.0 μm. *D*, flow cytometry–based quantification of Bodipy-C16 incorporation by Mtb isolated from pulse-labeled macrophages. Growth data (n = 4) ± SEM. Bodipy-C16 incorporation was quantified from 10,000 bacteria in each experiment by flow cytometry (n = 2) ± SD. Significance was calculated using one-way ANOVA with Dunnett’s multiple comparisons test (∗∗*p* < 0.01) ns = not significant. ΔMceG*, mceG* in Mtb; Mtb, *Mycobacterium tuberculosis.*

MceG has been primarily studied in the context of cholesterol import. Therefore, we sought to better characterize the role of MceG in the Mce1-mediated import of fatty acids. Free fatty acids are toxic and prevent the growth of Mtb in liquid culture, and this intoxication is mitigated by including albumin in the media during routine culturing (17). While the mechanism of fatty acid intoxication is unclear, we leveraged this intoxication phenotype as an indicator of fatty acid import. As expected, Mtb lacking the Mce1 fatty acid transporter was resistant to fatty acid intoxication in the absence of albumin while WT Mtb displayed poor growth in this assay (Fig. 2*A*). Similarly, Mtb lacking LucA, a protein that facilitates Mce1-mediated import of fatty acids, displayed a resistant phenotype in this growth assay (Fig. 2*A*). Mtb lacking MceG was resistant to fatty acid intoxication, while complementation restored the fatty acid intoxication phenotype (Fig. 2*B*). These observations further confirm that MceG is required for fatty acid import and fatty acids are toxic following import into the bacterial cell *via* the Mcel transporter.

### Fatty acid import in macrophages is mediated by MceG

Next, we assessed the role of MceG in fatty acid uptake during macrophage infection by quantifying bacterial assimilation of fluorescent palmitate (Bodipy-C16). In these experiments, Mtb was engineered to constitutively express mCherry and was used to infect murine macrophages. To track bacterial assimilation of Bodipy-C16 during infection in the macrophages, we pulse labeled the infected cells with the dye-labeled lipid. Following the pulse label, the bacteria were isolated from macrophages and analyzed by imaging and flow cytometry as described (8). Bodipy-C16 labeled, intrabacterial lipid inclusions were visualized in the WT and complemented strains. In contrast, bacteria lacking MceG produced little to no Bodipy-C16 lipid inclusions (Fig. 2*A*). Incorporation of Bodipy-C16 by intracellular Mtb was also quantified by flow cytometry revealing that, relative to WT and the complemented strain, we observed an ∼85% reduction in the amount of Bodipy-C16 assimilated by bacterial cells lacking MceG (Fig. 2*D*). These observations illustrate the essential role that MceG plays in importing fatty acids during macrophage infection.

### Analysis of protein abundance in the ΔMceG mutant

**Figure 3:**
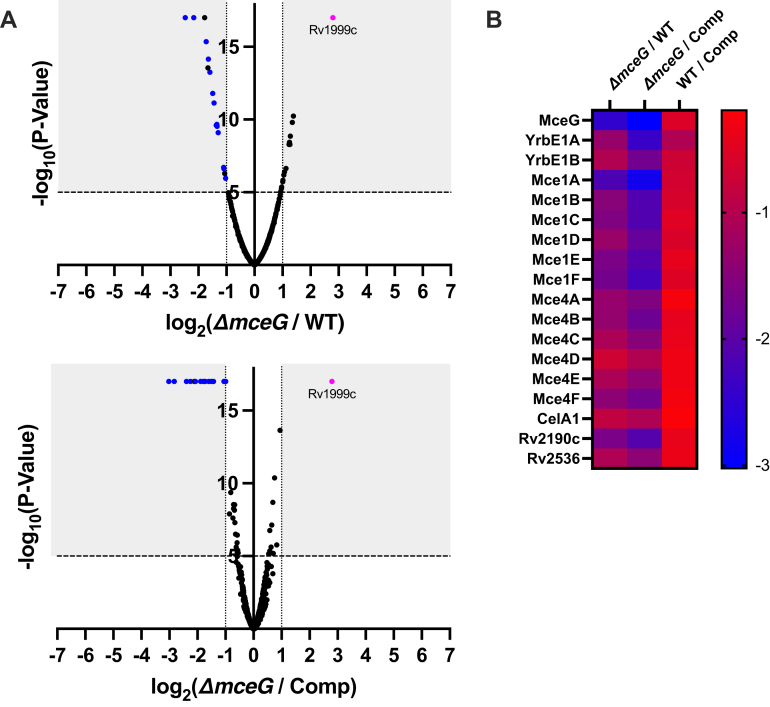
**Relative protein abundance in the ΔMceG mutant.***A*, abundance ratios were determined for 2429 proteins identified in proteomic analysis with coverage of two or more unique peptides coverage identified in the ΔMceG strain relative to WT (*top*) or the complemented strain (*bottom*). Shaded area indicates proteins with two-fold differences, *p* < 0.00001. *Blue dots* indicate Mce-associated proteins in the shaded regions of both plots, and *pink dot* indicates Rv1999c. *B*, heat map of proteins underrepresented in the ΔMceG strain relative to WT or the complemented strain with two-fold or greater differences and a *p* < 0.00001. Scale indicates log_2_ (fold change). ΔMceG*, mceG* in Mtb.

The Mce1 and Mce4 transporters are destabilized when Omam or LucA subunits are deleted. Thus, it is conceivable that Mce1 and Mce4 could also be destabilized in the ΔMceG mutant. To quantify protein turnover in Mtb lacking MceG, we employed an unbiased proteomic approach. For this, replicate cultures of Mtb (WT n = 5, ΔMceG n = 6, and complement n = 4) were grown to mid-log phase and lysed by sonication to generate SDS-solubilized whole cell lysates (WCLs). WCLs from each biological replicate were processed for tandem mass tag (TMT) labeling and protease digestion prior LC-MS/MS analysis. In total, this analysis identified 2429 proteins with a minimum of two unique peptides in each sample (Table S1). In our analysis, we applied a cut-off of log_2_(fold change), either more- or less-abundant, with a *p*-value < 0.00001 in the ΔMceG samples relative to WT or the complemented strain (Fig. 3*A*). Focusing on proteins common in both of these comparisons, we identified 18 underrepresented proteins in the ΔMceG samples. These were predominantly subunits of Mce transporters, including eight Mce1 proteins and five Mce4 proteins (Fig. 3*B*), supporting the idea that MceG stabilizes Mce transporters in Mtb. Recently, a cryo-EM structure of the *Mycobacterium smegmatis* Mce1 transporter was reported. Our proteomic approach identified homologs for the each of the core Mce1 proteins in Mtb including Rv2536/LucB (18). Importantly, Rv2536/LucB was previously not associated with the Mce1 transporter. One putative ABC transporter of the B-4DMT family, Rv2536, was also underrepresented in both data sets, suggesting that MceG may also stabilize and perhaps power this putative transporter. Conversely, one overrepresented protein, Rv1999c, was detected in the ΔMceG mutant relative to the WT and complement strains (Fig. 3*A*). Rv1999c is a putative integral membrane transporter of unknown function and may perhaps play a compensatory transport function in the absence of MceG, Mce1, and Mce4.

**Figure 4:**
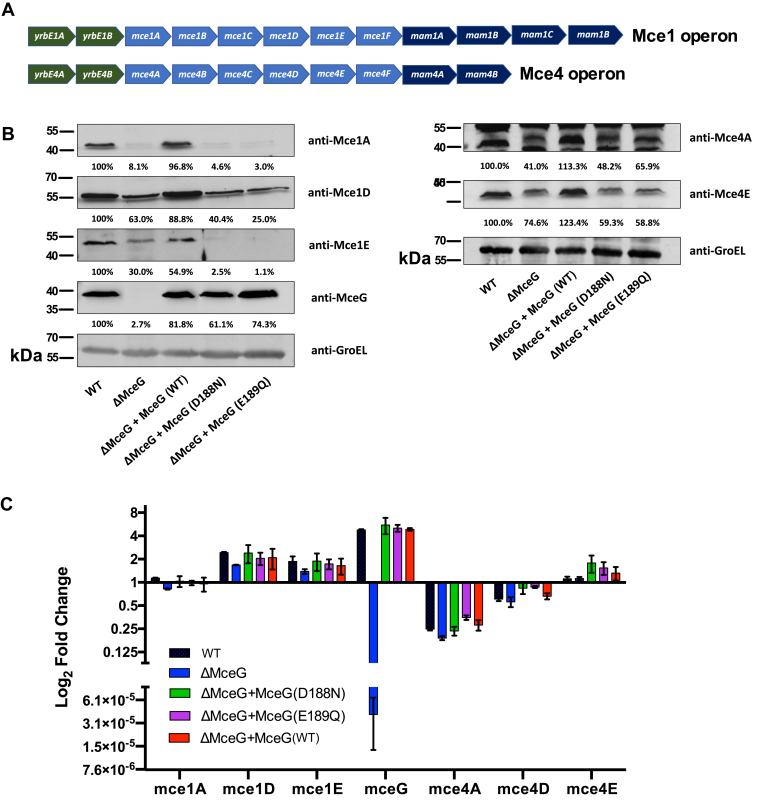
**MceG ATPase activity is required to stabilize the Mce1 transporter complex.***A*, genetic organization of the Mce1 and Mce4 operons. *B*, Mtb whole cell lysates probed with antibodies specific to protein of the Mce transporters and GroEL2 as the loading control. Western blots depicted are representative images of two independent replicates. Densitometry was performed with each Western blot and all values were normalized to those of the corresponding loading control. Ratios of the proteins levels of an individual lysate compared to the corresponding WT lysate are expressed as percentages inset below each blot. *C*, qPCR quantification of RNA transcripts from the Mce1 and Mce4 operons. Transcript levels were normalized to the housekeeping gene, *sigA*. Mtb, *Mycobacterium tuberculosis.*

Analysis of the bacterial WCLs by immunoblotting confirmed that the putative Mce1 substrate-binding proteins (Mce1A, Mce1D, and Mce1E) are degraded or less abundant in the ΔMceG mutant and in Mtb expressing the variants of MceG (D188N or E189Q) (Fig. 4*B*). To test if MceG and MceG ATPase activity is required to stabilize Mce proteins, bacterial cells were treated with chloramphenicol to prevent new protein synthesis, and protein decay of select Mce proteins was evaluated by Western blot. We found that the levels of Mce1D and the MceG (D188N) variant were reduced following chloramphenicol treatment (Fig. S2). Importantly, cells expressing WT MceG, the Mce1D, and MceG (D188N) variant protein remain stable and detectable in the presence of chloramphenicol treatment (Fig. S2). Mce protein turnover in the absence of any detectable gene expression differences is considered a property of Mce transporter destabilization in Mtb (8, 13). Quantification of *mceG*, *mce1*, and *mce4* transcript levels using qPCR confirmed that gene expression of these genes and operons is equivalent between all strains, and depletion of Mce1 and Mce4 proteins is not due to a reduction in gene expression (Fig. 4*C*). These data provide evidence that MceG, and, more specifically residues in the Walker B motif of MceG plays a role in stabilizing the components of the Mce1 and Mce4 transporter complexes. While protein concentration in cells is a product of synthesis and degradation, these data suggest that Mce proteins are subject to decay when MceG is deleted or lacks critical ATPase active site residues.

### MceG exhibits ATPase activity

Importantly, the enzymatic activity of MceG has not yet been experimentally demonstrated. Therefore, we sought to detect ATPase activity from MceG. To do this, WT MceG and a version of MceG containing a mutated Walker B motif (D188N) were each fused to an N-terminal His-SUMO tag to enhance solubility and purification from *Escherichia coli.* The recombinant proteins were purified to homogeneity and analyzed for ATP hydrolysis activity. The effective activity for WT MceG was 0.198 ± 0.007 P_i_ min^−1^ (Fig. S3). The MceG (D188N) variant exhibited background ATP hydrolysis activity (0.096 ± 0.002 P_i_ min^−1^), which is likely due to partial inactivation of the enzyme or a contaminating ATPase carried over from the purification process (Fig. S3). This low but detectable ATPase activity with WT MceG likely reflects a requirement for additional protein components of the Mce transporters for full MceG ATPase activity similar to what has been observed with the related *E. coli* Mla transporter (19, 20).

### MceG is required for full fitness in murine lung tissue

**Figure 5:**
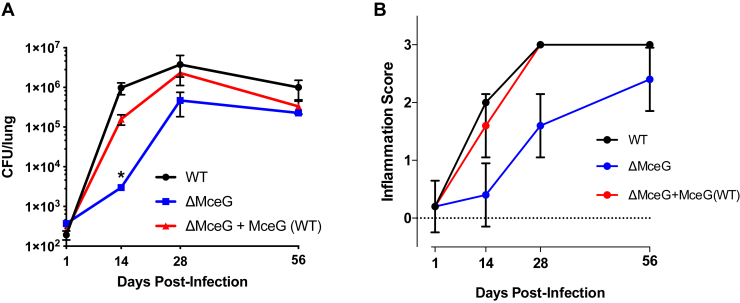
**MceG is required for Mtb survival *in vivo***. *A* and *B*, BALB/c mice were infected by intranasal inoculation using similar numbers of bacteria. *A*, colony forming units of WT, ΔMceG, and ΔMceG+MceG(WT) bacteria from the lungs of mice individually plotted. *B*, histopathology as scored by a pathologist in a blinded manner. Score numbers represent the extent of inflammatory lesions: 0, no lesions; 1, mild inflammation; 2, moderate inflammation; 3, marked inflammation. Data represent two or more independent experiments. Data are means ± SEM. Significance was calculated using Mann-Whitney test (∗*p* < 0.05). Mtb, *Mycobacterium tuberculosis*; ΔMceG*, mceG* in Mtb.

The *in vivo* fitness of an MceG mutant has only been reported from mice using an intravenous, competitive infection assay (11). Mtb mutants lacking LucA are unable to import both cholesterol and fatty acids and have a colonization defect in the lungs of mice (8). Therefore, we characterized the MceG mutant using the same infection model and found that the ΔMceG mutant grew poorly during the first 2 to 3 weeks in mice resulting in a ∼0.5 to 1.0 log_10_ reduction in colony-forming units (CFUs) across the remainder of the infection (Fig. 5*A*). The decrease in bacterial CFUs correlates with a reduction in the levels of inflammation in the lungs (Fig. 5*B*).

## Discussion

The Mce1 and Mce4 transporters appear to be comprised of core substrate-specific proteins but also share specific subunits such as MceG, LucA, and Omam proteins (8, 12, 13). This is a highly unusual arrangement for a transporter family and how all of these proteins coordinate with the different Mce transporters remains unknown. Transporter ATPase subunits do mechanical work by undergoing conformational changes following ATP hydrolysis by transferring energy to coupling helices of permease proteins during substrate translocation (21). Each of the four Mce transporters in Mtb is thought to consist of two integral membrane permease proteins, termed YrbE1-4A and YrbE1-4B. All eight of these putative permease proteins have similar predicted transmembrane topologies with a homologous, 47 amino acid cytosolic loop containing an EExDA motif which is a strong candidate for a shared MceG-binding site analogous to a coupling helix (10). While MceG may also stabilize Mce2 and Mce3 in Mtb, we did not reliably detect depletion of these proteins; this is partly because of their limited expression under the growth condition used (22, 23).

Low levels of MceG ATPase activity were detected using recombinant MceG alone, and full ATPase activity will likely require purifying MceG in a complex with additional Mce transporter proteins. The MceG Walker A domain is thought to coordinate ATP binding *via* hydrogen bonding with β-phosphate, and the Walker B domain could provide the carboxylate residue needed to coordinate Mg^2+^ for ATP hydrolysis (24). We focused on mutating the catalytic residues in the Walker B motif of MceG because chaperone ATPases lacking these residues can bind ATP but cannot hydrolyze it (25). The Walker B mutations in MceG were sufficient to destabilize Mce1 and Mce4, suggesting ATP hydrolysis is also needed to maintain Mce1 and Mce4 stability and/or function. A recent report describes a stability-based mechanism thought to regulate the *E. coli* Mla transporter, which facilitates movement of phospholipids across the periplasm and utilizes an ABC ATPase, MlaF, to energize this process (26). In this model, a soluble STAS-domain–containing protein, MlaB, is proposed to regulate the Mla transporter by stabilizing the MlaF dimer, either by preventing its degradation or by increasing affinity of MlaF for the transporter (19). It is possible that additional factors such as LucA or the Omam proteins may be required to facilitate the binding of MceG to the Mce transporters, leading to stabilization of the Mce transporters.

Free fatty acids are known to intoxicate bacteria by disrupting membrane functions (27, 28) or inducing lipid peroxidation reactions in cells (29). It is well established that Mtb can be intoxicated by fatty acids in liquid media that lacks carrier proteins such as albumin (17, 30, 31). Complexing free fatty acids to albumin in media formulations likely limits Mtb’s ability to import and/or metabolize these substrates. While the mechanism of fatty acid intoxication in Mtb remains unclear, this intoxication phenotype correlates with Mtb’s ability to import fatty acids suggesting an intoxication process that occurs following delivery of the fatty acids into the Mtb cell.

In our proteomic analysis, the Mce1 and Mce4 transporter proteins are the most underrepresented proteins in ΔMceG mutant, with the exception of three proteins (Rv2536, Rv2190/RipC, and Rv0062/CelA1) (Fig. 3*B*). The function of Rv2536 is unknown, but peptides from this putative membrane protein bind mammalian cell surfaces with high affinity (32), a property similar to other Mce proteins (33). Rv2190/RipC is a member of the NlpC/P60 family of proteins that hydrolyze proteins, peptidoglycan fragments, and catalyze acyl transfer reactions (34). *In vitro*, Rv2190/RipC has weak but detectable peptidoglycan hydrolase activity which is stimulated by binding to the cell division protein, FtsX (35). The role of Rv2190/RipC in Mtb cell division is unclear but mutants lacking Rv2190/RipC are attenuated in the TB mouse model and have altered cell morphology associated with cell wall integrity defects (36). While we did not observe any obvious colony morphology differences in the ΔMceG mutant, we cannot exclude that some of the *in vivo* phenotype of ΔMceG observed could also reflect depletion of Rv2190/RipC. Our future work will determine whether Rv2190/RipC is involved in the turnover of the Mce1 and Mce4 transporters. Lastly, Rv0062/CelA1 is a putative secretory protein with cellulase activity (37) that is capable of degrading the types of extracellular carbohydrates produced by Mtb (38). These results suggest that there is a dynamic remodeling of the Mtb cell wall to accommodate Mce transporter function or to maintain the cell wall in the absence of these transport complexes.

Several practical challenges exist that make targeting nutrient utilization in Mtb for drug development difficult. Mtb can simultaneously import and metabolize various nutrients and seemingly lacks a canonical catabolite repression system (39). Mtb also colonizes various tissues and host cells during infection and is therefore thought to be exposed to a variety of different nutrient types in its natural environment (3, 40, 41). Additionally, the lipid catabolic pathways in Mtb are likely highly redundant and true rate-limiting bottlenecks in these pathways are only beginning to be understood (42). However, within macrophages, Mtb encounters some nutritional constraints that impose a preference for the bacteria to metabolize fatty acids and cholesterol (43). Therefore, chemically corrupting cholesterol and fatty acid import in Mtb could negatively impact bacterial fitness during infection in macrophages or lipid-rich environments of necrotic granulomas. This work suggests that effective chemical inhibitors of MceG would be predicted to block Mtb’s ability to utilize multiple key lipid nutrients simultaneously while negatively impacting bacterial fitness, which may enhance antibiotic treatment options for TB.

## Experimental procedures

### Strains and growth conditions

The *E. coli* strains Top10 (Invitrogen) and T7 Express (New England Biolabs) were used for molecular cloning. *E. coli* strains were grown in LB medium and transformants were selected on LB plates containing kanamycin (25 μg ml^−1^), ampicillin (100 μg ml^−1^), or hygromycin (100 μg ml^−1^). For protein expression, the *E. coli* strain BL21 (DE3) (Stratagene) was used, and strains were grown in LB medium or Terrific Broth media containing antibiotics. Mtb Erdman strains were cultivated in 7H9 medium (BD Biosciences) containing OADC supplement (BD Biosciences) unless otherwise noted. 7H12 base medium was used as previously described (43) and supplemented with cholesterol (100 μM), fatty acids (100 μM), or sodium acetate (0.1%). Stock solutions (1000×) of lipid substrates were solubilized in a (1:1 v/v) solution of tyloxapol (Sigma) and ethanol and heated to 65 °C prior to adding into media to the final concentration of tyloxapol at 0.05%. The Δ*mce1* and Δ*lucA* mutant strains were generated in (8). To create the Δ*mceG* mutant strain, the internal region of the *rv0655* ORF(111 bp −499 bp) was replaced with a hygromycin cassette *via* allelic exchange and confirmed with sequencing (44). For fatty acid toxicity studies, Mtb strains were cultured to mid-log phase in 7H9 OADC + 0.05% tyloxapol and then inoculated at an A_600_ of 0.005 into 10 ml of 7H12 media lacking casitone and supplemented with 25 μM palmitate. Cultures were incubated at 37 °C for 21 days and an A_600_ measurement was taken every 3 to 4 days over the course of the incubation period.

### Protein expression and purification

To express recombinant MceG, a plasmid containing a truncated version of WT or mutant MceG fused at the N-terminus to a His_6_-SUMO tag were transformed into *E. coli* BL21 (DE3) cells. To generate MceG point mutants, site-directed mutagenesis was performed using the QuickChange method (Agilent). For protein expression, these strains were cultured in 18.0 L of Terrific Broth medium containing kanamycin (50 μg ml^−1^) to an A_600_ of 0.6 at 37 °C. The temperature was then lowered to 18 °C, and 0.5 mM IPTG (Sigma) was added to induce gene expression. Cultures were incubated for 16 h at 18 °C and shaking at 220 rpm. Cells were harvested by centrifugation and sonicated in buffer A (25 mM Tris–HCl, pH 8.5, 500 mM NaCl, 20 mM imidazole). Centrifugation was used to remove debris, and the His-tagged proteins were affinity purified using Ni-nitrilotriacetic acid resin. Protein was eluted off the resin with buffer B (25 mM Tris–HCl, pH 8.5, 500 mM NaCl, 500 mM imidazole). A HiPrep 26/10 Desalting column (GE Life Sciences) was used to buffer exchange the protein into gel filtration buffer (25 mM Tris–HCl, pH 7.5, 150 mM NaCl), and it was further purified using an S200 size-exclusion chromatography column (GE). Purified protein from peak fractions was concentrated to 10 mg ml^−1^ and stored at −80 °C. Protein purity was assessed *via* SDS-PAGE and Coomassie staining.

### ATPase assay

ATPase activity of recombinant WT or mutant MceG was measured using the Enzchek Phosphate Assay kit (Invitrogen). A reaction mixture was made following the kit instructions and scaled to reaction volume of 200 μl. The reaction mixture was added to a 96-well plate along with recombinant WT or mutant MceG at concentrations of 2.5, 5, 12.5, and 25 μM. Following the addition of 0.5 mM ATP, inorganic phosphate accumulation was assessed over time through measurement of the absorbance at 360 nm using an Envision plate reader (PerkinElmer).

### Lipid uptake assays

Lipid uptake was quantified as described (8) with slight modifications. Briefly, Mtb was cultured in vented T-25 tissue culture flasks with 7H9 base media supplemented with 0.5% bovine serum albumin Fraction V, 0.2% dextrose, 0.01% glycerol, and 0.05% tyloxapol for 5 days. At the mid-log phase of growth, the bacteria were harvested and the cell density was adjusted to an A_600_ of 0.7 in 7.0 ml of spent medium and were incubated with 1 μCi of [^14^C(U)]-palmitate (PerkinElmer) or [4-^14^C] cholesterol (PerkinElmer) at 37 °C for 2 h. Bacterial samples (1.5 ml) were removed at 5, 30, 60, and 120-min time points, washed three times in 1 ml of ice-cold wash buffer (0.1% fatty acid free-BSA and 0.1% Triton X-100 in PBS), and fixed in 0.2 ml of 4% paraformaldehyde for 1 h. The total amount of radioactive label associated with the fixed pellet was quantified by scintillation counting. The radioactive signal was normalized to the relative levels of bacterial density at A_600_ for the bacterial cultures before addition of radioactive label. The uptake rate was calculated by applying linear regression to the normalized radioactive counts over time, and uptake efficiency was expressed as a ratio of uptake rate for each strain relative to the WT control. The rate of lipid uptake calculated for each strain was then normalized to WT in order to express the rate as uptake efficiency (%). For sodium azide experiments, flasks were inoculated with sodium azide at a final concentration of 60 mM and incubated at room temperature for 10 min before radiolabel was added.

### Radiorespirometry assays

Lipid oxidation was monitored by quantifying the release of ^14^CO_2_ from [4-^14^C]-cholesterol or [^14^C(U)]-palmitate by radiorespirometry. Mtb cultures were pregrown in 7H9 base media supplemented with 0.5% BSA Fraction V, 0.2% dextrose, 0.01% glycerol, and 0.05% tyloxapol for 5 days. At the mid-log phase of growth, the density of the bacterial cells was adjusted to an A_600_ of 0.7 in 5 ml of spent medium supplemented with 1.0 μCi of radiolabeled substrates in vented standing T-25 tissue culture flasks placed in a sealed air-tight vessel with an open vial containing 0.5 ml 1.0 M NaOH at 37 °C. After 5 h, the NaOH vial was recovered, neutralized with 0.5 ml 1.0 M HCl, and the amount of base soluble Na_2_^14^CO_3_ was quantified by scintillation counting. Radioactive counts were normalized to the relative levels of bacterial growth by determining the A_600_ for the bacterial cultures obtained at the 5 h timepoint. The percentage of CO_2_ release was expressed as a ratio of normalized radioactive signal for each strain relative to the WT control as described (8).

### Macrophage isolation and fluorescent fatty acid import assay

Macrophages were derived from the bone marrow from femurs of BALB/c mice (Jackson), 6 to 8 weeks of age (45). The bone-marrow–derived macrophages were seeded into T-150 tissue culture flasks (3 × 10^7^ cells per flask) and infected with Mtb at a multiplicity of infection of 4:1. After 3 days of infection, Bodipy-16 (Thermo Fisher Scientific) to a final concentration 8 μM preconjugated to defatted 1% BSA was added to the cells for 1-h pulse and then chased with cell media for another hour. The infected macrophages were scraped into 15 ml of homogenization buffer (250 mM sucrose, 0.5 mM EGTA, 20 mM Hepes, 0.5% gelatin, pH 7.0) and pelleted by centrifugation at 514 × G (1500 rpm, Beckman Allegra 6KR centrifuge, GH-3.8 rotor), followed by cell lysis by 70 passages through a 25-gauge needle. Five milliliters of cell lysate were centrifuged at 146 × G (800 rpm) for 10 min; supernatant (suspensions of phagosomes) was retained and treated with 0.1% Tween-80 at 4 °C for 15 min to lyse Mtb-containing vacuoles. Isolated bacteria were washed once in PBS + 0.05% tyloxapol and fixed in 4% PFA. Flow cytometry data were collected on BD FACS LSR II and analyzed using FlowJo (Tree Star, Inc).

### Microscopy and image analysis of lipid inclusions

Monolayers of macrophages infected at an MOI of 3:1 in 8-well glass bottom μ-Slides (Ibidi) were pulsed with 8 μM Bodipy-C16 (Thermo Fisher Scientific) complexed to fatty acid–free BSA as described (8, 12, 46). Following the pulse labeling period, the cells were chased in fresh media without label for 1 h. The infected macrophages were imaged by confocal microscopy as described (47).

### Quantitative LC-MS/MS proteomics

*M. tuberculosis* cultures were cultured in 50 ml of 7H9 OADC + 0.05% tyloxapol standing for 5 days. Bacteria were suspended in 1× PBS containing 1% SDS + protease inhibitors and lysed *via* sonication on ice. Samples were centrifuged for 10 min at 15,000 RPM. The supernatant was filtered, then submitted to Cornell Proteomics and Metabolomics Facility. Protein concentration for each sample was determined by running on a precast NOVEX 10% Bis-Tris mini-gel (Invitrogen) with serial amounts of *E. coli* lysates (2.5, 5, 10, 15 μg/lane) serving as a standard curve. The SDS gel was visualized with colloidal Coomassie blue stain (Invitrogen), imaged by ChemiDoc (Bio-Rad) followed by quantification using Image Lab 6.1 (Bio-Rad).

Proteins were digested and labeled according to Thermo Fisher Scientific’s TMTpro Mass Tagging Kits and Reagents protocol (Lot number: VL313890) with slight modifications (48, 49, 50, 51). A total of 25 μg protein of each sample were suspended in 50 mM triethylammonium bicarbonate (TEAB) pH 8.5, 6M urea, 2M thiourea, 1% SDS and reduced with 10 mM Tris(2-carboxyethyl) phosphine for 1 h at 34 °C, alkylated with 20 mM iodoacetamide for 45 min in the dark, and then quenched with a final concentration of 32 mM DTT. Each sample was digested separately using the S-Trap Micro Spin column (ProtiFi) (52). After quenching, 12% phosphoric acid was added to a final concentration of 1%, followed by 1:7 dilution (v/v) with 90% methanol, 0.1 M TEAB pH 8.5. The samples were loaded into an S-Trap Micro spin column and centrifuged at 4000g for 30 s, then washed three times with 150 μl of 90% methanol, 0.1 M TEAB pH 8.5. Digestion was performed with 25 μl trypsin at 100 ng/μl (1:10 w/w) in 50 mM TEAB pH 8.5 added to the top of the spin column. Spin columns were incubated overnight (16 h) at 37 °C. Following incubation, the digested peptides were eluted off the S-trap column sequentially with 40 μl each of 50 mM TEAB pH 8.5, 0.2% formic acid, 50% acetonitrile (ACN)-0.2% formic acid. Three eluates were pooled together and dried before reconstituting in 100 μl water and redried to remove residual formic acid.

Prior to labeling, each sample was reconstituted into 30 μl 0.1 M TEAB pH 8.5. The TMT16-plex label was reconstituted with 15 μl of anhydrous ACN prior to labeling, added to each of the 30 μl tryptic digest samples (1:6.6 w/w ratio peptide to TMT label), and incubated for 1 h at room temperature. The labeled peptides from the 15 samples were pooled together. The pooled peptides from each replicate were then evaporated to dryness and cleanup by solid phase extraction on MCX Cartridges (Waters). Labeling incorporation was checked using Orbitrap Eclipse Thermo Fisher Scientific). The eluted tryptic peptides were evaporated to dryness and fractionated using a Dionex UltiMate 3000 HPLC system with the built-in micro fraction collection option in its autosampler and UV detection (Thermo Fisher Scientific) as described (48, 49, 50, 51). The TMT-labeled tryptic peptides were reconstituted in buffer A (20 mM ammonium formate pH 9.5 in water) and loaded onto an XTerra MS C18 column (3.5 μm, 2.1x 150 mm) from Waters, with 20 mM ammonium formate (NH_4_FA), pH 9.5 as buffer A and 80% ACN/20% 20 mM NH_4_FA as buffer B. The LC was performed using a gradient from 10 to 45% of buffer B in 30 min at a flow rate of 200 μl/min. Forty eight fractions were collected at 1-min intervals and pooled into a total of 10 fractions based on the UV absorbance at 214 nm with multiple fraction concatenation strategy (53). Each of the 10 fractions was dried and reconstituted in 100 μl of 2% ACN/0.5% formic acid for nanoLC-MS/MS analysis.

The nanoLC-MS/MS analysis was carried out using an Orbitrap Eclipse (Thermo Fisher Scientific) mass spectrometer equipped with a nanospray Flex Ion Source coupled with the UltiMate 3000 RSLCnano (Dionex). Each reconstituted fraction (3.5 μl = 0.7 μg for global proteomics fractions) was injected onto a PepMap C-18 RP nano trap column (5 μm, 100 μm × 20 mm, Dionex) at 20 μl/min flow rate for rapid sample loading and separated on a PepMap C-18 RP nano column (2 μm, 75 μm ×25 cm). The column was equilibrated with 2% ACN in 0.1% aqueous formic acid (eluant A) prior to each run. The labeled peptides were eluted in a 120 min gradient of 5% to 32% eluant B containing 95% ACN in 0.1% formic acid at 300 nl/min, followed by an 8-min ramping to 90% B, a 7-min hold, and 21-min re-equilibration with 2% ACN-0.1% FA prior to the next run. The Orbitrap Eclipse was operated in positive ion mode with nanospray voltage set at 1.9 kV and source temperature at 300 °C. External calibration for FT, IT, and quadrupole mass analyzers was performed. Raw MS data files for all the fractions were acquired using a real-time search (RTS) synchronous precursor selection MS^3^ workflow as reported previously (48). Specifically, the RTS MS^3^ workflow consisted of 2.5 s “Top Speed” data-dependent collisionally induced dissociation-MS/MS scans (for peptide identifications by RTS) that enabled to trigger synchronous precursor selection of 10 MS^2^ product ions for subsequent MS^3^ in FT. In RTS mode, the *M. tuberculosis* NCBI database downloaded November 2021 that contains 417,575 sequences, which were imported as the FASTA database with trypsin as the enzyme for real-time spectral database search respectively for the samples from corresponding species. The search parameters included the following: TMTpro modifications on lysine and N-terminal amines (Δmass 304.2071), carbamidomethyl modification of cysteine (Δmass 57.0215), and maximum two variable methionine oxidation per peptide, and one missed cleavage. A maximum search time for 35 ms allowed for the RTS MS^3^ searching. The MS^3^ scan was carried out using a mass range of 110 to 500 m/z, an MS isolation window of 1.1 m/z, and MS^2^ isolation window of 2.0 m/z were used. A resolving power of 50,000 at MS^3^ with a normalized collision energy of 55% was used for peptide quantitation. Other parameters included 200% normalized AGT target and 120 ms for maximum injection time. Dynamic exclusion parameters were set at 1 within 50 s exclusion duration with ±10 ppm exclusion mass window. All data were acquired under Xcalibur 4.3 operation (https://www.thermofisher.com) software in Orbitrap Eclipse (Thermo Fisher Scientific).

### Data processing, protein identification, and data analysis

All raw MS spectra were processed and searched using the Sequest HT search engine within the Proteome Discoverer 2.5 (PD 2.5, Thermo Fisher Scientific). A *M. tuberculosis* strain Erdman ATCC 35801 NCBI database comprised of 4222 sequences was used for post-MS database searches. The default search settings used for 16-plex TMT quantitative processing and protein identification in PD 2.5 searching software (https://www.thermofisher.com) were as follows: two mis-cleavages for full trypsin with fixed carbamidomethyl modification of cysteine, fixed 16-plex TMT modifications on lysine and N-terminal amines along with variable modifications of methionine oxidation, deamidation on asparagine/glutamine residues and protein N-terminal acetylation. The peptide mass tolerance and fragment mass tolerance values were 10 ppm for MS survey scan, 0.6 Da for MS^2^, and 20 ppm for MS^3^, respectively. Identified peptides were further filtered for maximum 1% false discovery rate using the Percolator algorithm in PD 2.5 along with additional peptide confidence set to high and peptide mass accuracy ≤5 ppm. The TMT16-plex quantification method within Proteome Discoverer 2.5 software was used to calculate the reporter ion abundances in MS^3^ spectra that were corrected for the isotopic impurities. Both unique and razor peptides were used for quantitation. Signal-to-noise values were used to represent the reporter ion abundance with a coisolation threshold of 50% and an average reporter signal-to-noise (intensity) threshold of ≥10 used for quantitation spectra. The intensities of peptides, which were summed from the intensities of the peptide-spectrum match, were summed to represent the abundance of the proteins. For relative ratio between the two groups, normalization on total peptide amount for each sample was applied. *t* test was used for *p*-values calculation of the reported ratios. The search result including ratio and peptide abundance for each sample was provided as an output to Microsoft Excel software for further data analysis.

### Western blot analysis

To obtain WCLs for Western blot analysis, Mtb strains were cultured in 40 ml of 7H9 OADC + 0.05% tyloxapol to an A_600_ of 0.6. Bacteria were harvested through centrifugation and fixed for 1 h with 4% PFA. Cells were washed with PBS 0.05% tyloxapol and lysed in 1% SDS using sonication. Where applicable, 20 μg/ml chloramphenicol was added 2 days prior to harvesting. After separation *via* SDS-PAGE, proteins were transferred to a nitrocellulose membrane. Antibodies for GroEL were obtained from BEI resources and anti-MceG antibodies were generated as previously described (8). Antibodies for Mce1A, Mce1D, and Mce1E were a gift from Christopher Sassetti (54). Antibodies for Mce4A and Mce4E were a gift from Miriam Braunstein (14).

### Isolation of RNA and qPCR analysis

RNA was purified from WT, *mceG* mutant, complement, D188N or E189Q complement *Mtb* growing in exponential phase. Bacteria were pelleted and lysed in Trizol LS (Invitrogen) with 0.1 mm silica beads in a FastPrep-24 bead beating grinder (MP Biomedicals). The RNA was purified using Trizol LS per manufacturer instructions with the addition of a second chloroform extraction and a second ethanol wash. The DNA was digested using TURBO DNA-free Kit (Invitrogen).

For relative quantification of gene expression, complementary DNA was generated from 250 ng of RNA using iScript complementary DNA synthesis kit (Bio-rad), and real-time PCR was performed using the iTaq SYBR Green kit (Bio-rad) on the 7500 Fast Real-Time PCR System (Applied Biosystems). Gene-specific primers were designed using Primer3 software (https://primer3.ut.ee/). The sigma factor gene *sigA* (*rv2703*) was used to normalize each sample, and approximate fold induction compared to WT was calculated using the 2^-ΔCT^ method (55). The average and range of fold induction were calculated using the average and SD of ΔCT from four experimental replicates, and statistical changes in ΔCT were determined using two-way ANOVA and Dunnett’s multiple comparisons (56).

### Mouse infection studies

Animal work was approved by Cornell University IACUC (protocol number 2013-0030). All protocols conform to the USDA Animal Welfare Act, institutional policies on the care and humane treatment of animals, and other applicable laws and regulations. Six to eight-week-old female BALB/cJ WT mice Jackson Laboratories were infected with 1000 CFU of Mtb strains *via* an intranasal delivery method as described (57). The mice were anesthetized with isoflurane and 25 μl of bacteria were introduced into both nares. At sacrifice, the lungs were removed and half of the lungs were fixed in 4% PFA overnight for histology studies, while another half was used for bacterial load quantification. For the latter, lungs were homogenized in PBS 0.05% Tween-80 and plated on 7H10 OADC agar. CFU were quantified after 3 to 4 weeks incubation at 37 °C.

## Data availability

All data are contained within the article. Material described is available upon request from the corresponding author.

## Supporting information

This article contains supporting information.

## Conflicts of interest

All authors declare no conflicts of interest with the contents of this article.
