# Supplementary figures and images for: MceG stabilizes the Mce1 and Mce4 transporters in Mycobacterium tuberculosis
Source: J Biol Chem. 2023 Jan 13;299(3):102910. doi: 10.1016/j.jbc.2023.102910 (PMC9947336; doi:10.1016/j.jbc.2023.102910)

**A**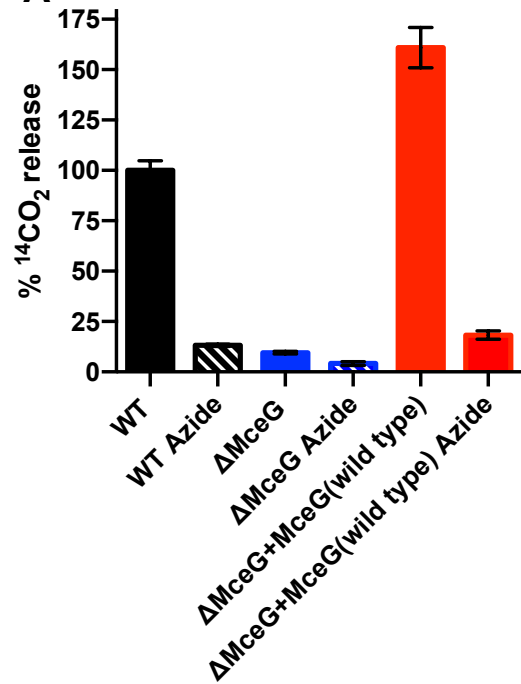**B**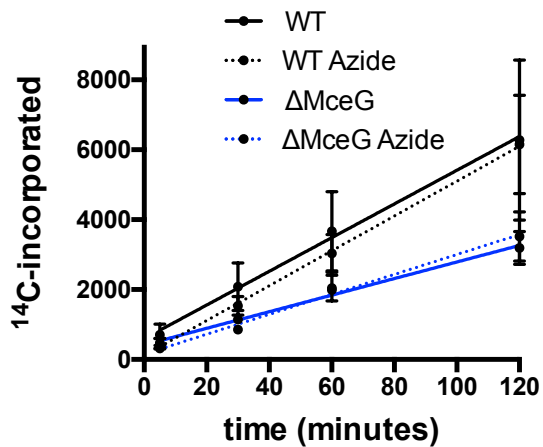**C**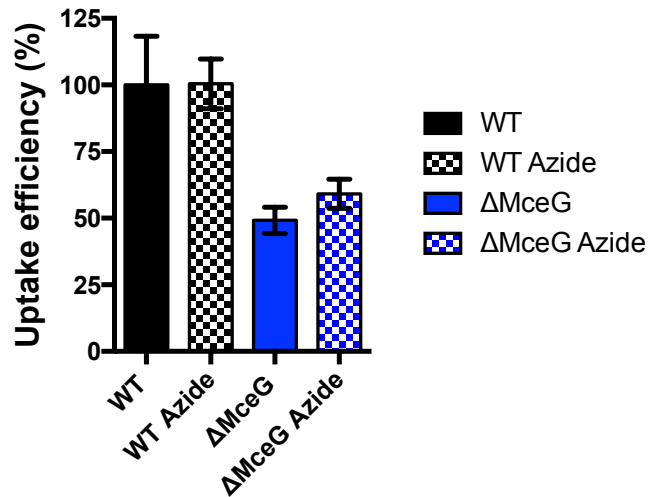

Supplement: Figure S1 [file mmc2.pdf]

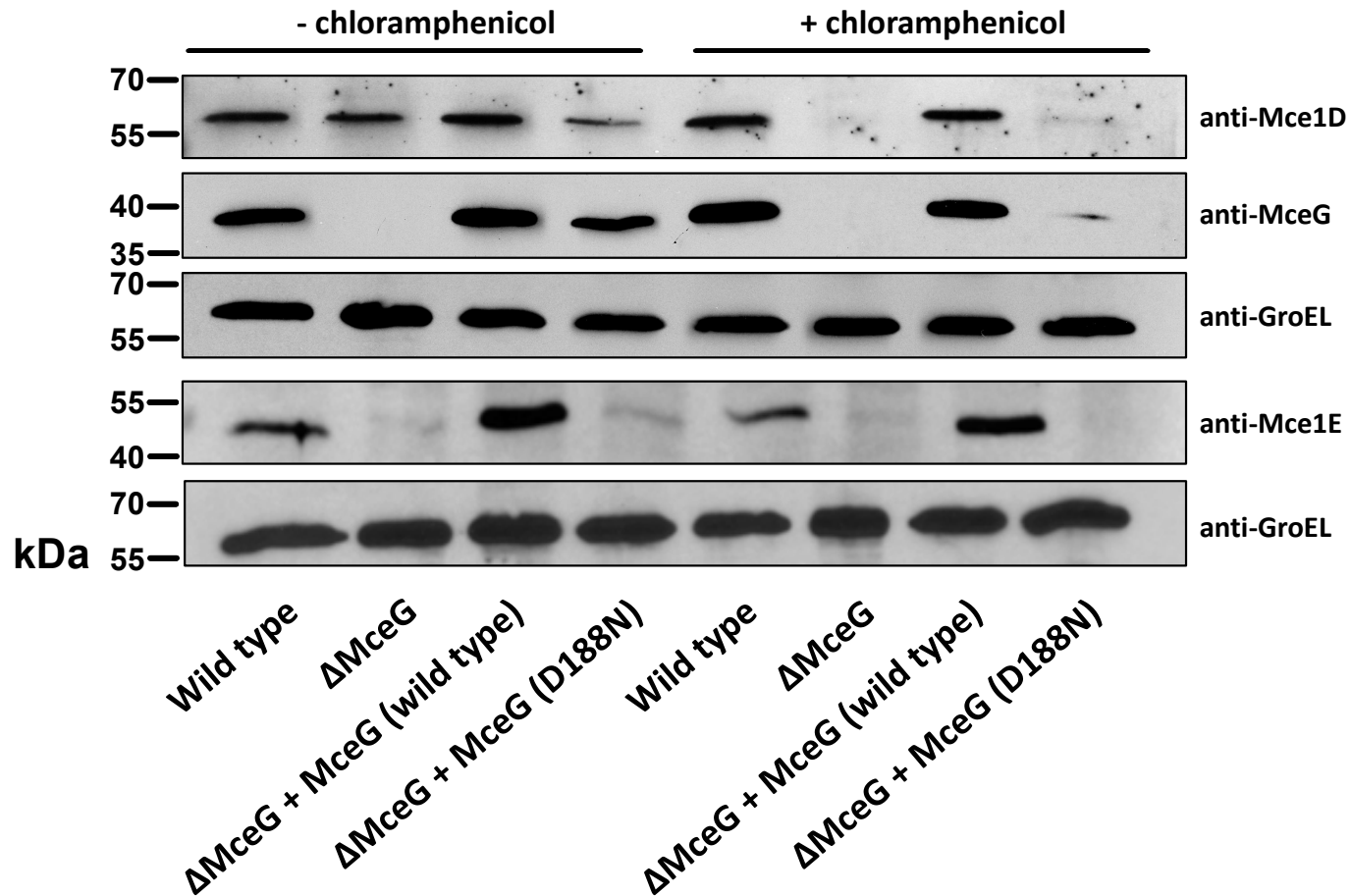

Supplement: Figure S2 [file mmc3.pdf]

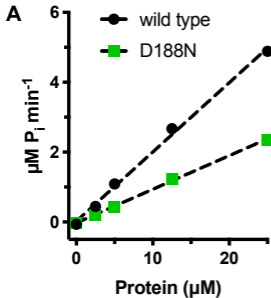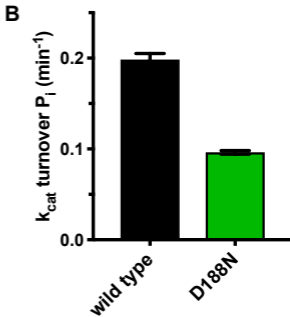

Supplement: Figure S3 [file mmc4.pdf]
